# The Microbiology of Bloodstream Infection: 20-Year Trends from the SENTRY Antimicrobial Surveillance Program

**DOI:** 10.1128/AAC.00355-19

**Published:** 2019-06-24

**Authors:** Daniel J. Diekema, Po-Ren Hsueh, Rodrigo E. Mendes, Michael A. Pfaller, Kenneth V. Rolston, Helio S. Sader, Ronald N. Jones

**Affiliations:** a University of Iowa, Iowa City, Iowa, USA; b National Taiwan University Hospital, National Taiwan University College of Medicine, Taipei, Taiwan; c JMI Laboratories, North Liberty, Iowa, USA; d The University of Texas MD Anderson Cancer Center, Houston, Texas, USA

**Keywords:** antimicrobial resistance, bloodstream infection, surveillance

## Abstract

Bloodstream infection (BSI) organisms were consecutively collected from >200 medical centers in 45 nations between 1997 and 2016. Species identification and susceptibility testing followed Clinical and Laboratory Standards Institute broth microdilution methods at a central laboratory. Clinical data and isolates from 264,901 BSI episodes were collected. The most common pathogen overall was Staphylococcus aureus (20.7%), followed by Escherichia coli (20.5%), Klebsiella pneumoniae (7.7%), Pseudomonas aeruginosa (5.3%), and Enterococcus faecalis (5.2%). S. aureus was the most frequently isolated pathogen overall in the 1997-to-2004 period, but E. coli was the most common after 2005. Pathogen frequency varied by geographic region, hospital-onset or community-onset status, and patient age. The prevalence of S. aureus isolates resistant to oxacillin (ORSA) increased until 2005 to 2008 and then declined among hospital-onset and community-acquired BSI in all regions. The prevalence of vancomycin-resistant enterococci (VRE) was stable after 2012 (16.4% overall). Daptomycin resistance among S. aureus and enterococci (DRE) remained rare (<0.1%). In contrast, the prevalence of multidrug-resistant (MDR) Enterobacteriaceae increased from 6.2% in 1997 to 2000 to 15.8% in 2013 to 2016. MDR rates were highest among nonfermentative Gram-negative bacilli (GNB), and colistin was the only agent with predictable activity against Acinetobacter baumannii-Acinetobacter calcoaceticus complex (97% susceptible). In conclusion, S. aureus and E. coli were the predominant causes of BSI worldwide during this 20-year surveillance period. Important resistant phenotypes among Gram-positive pathogens (MRSA, VRE, or DRE) were stable or declining, whereas the prevalence of MDR-GNB increased continuously during the monitored period. MDR-GNB represent the greatest therapeutic challenge among common bacterial BSI pathogens.

## Abstract (precis)

Bloodstream infection (BSI) organisms were consecutively collected from >200 medical centers in 45 nations between 1997 and 2016. Species identification and susceptibility testing followed Clinical and Laboratory Standards Institute broth microdilution methods at a central laboratory.

## INTRODUCTION

Bloodstream infection (BSI) causes significant patient morbidity and mortality worldwide (1). Changing antimicrobial resistance (AMR) rates, pathogen distribution, demographics, and medical care delivery all may affect the epidemiology of BSI; therefore, continuously monitoring trends in the microbiology of BSI pathogens worldwide is very important. Examining microbiological trends can help inform diagnostic approaches, treatment strategies, and prevention programs.

A major concern has been the emergence and global spread of multidrug-resistant (MDR) organisms, including oxacillin-resistant Staphylococcus aureus (ORSA), vancomycin-resistant *Enterococcus* spp. (VRE), and MDR Gram-negative bacilli (GNB) (including extended-spectrum-β-lactamase [ESBL] producers), carbapenem-resistant Enterobacteriaceae (CRE), and MDR nonfermenters such as Pseudomonas aeruginosa and Acinetobacter spp. Several studies have demonstrated the high mortality attributable to BSI due to these MDR organisms (2–5).

The SENTRY Antimicrobial Surveillance Program was established in 1997 to monitor the predominant bacterial pathogens and the antimicrobial resistance patterns of organisms isolated from patients with various infection types, including BSI (6). We now report trends in organism distribution and AMR among BSI isolates submitted to the SENTRY Program during the first 20 years of the program (1997 to 2016).

## RESULTS

Among the 264,901 BSI isolates collected, the most common pathogens overall were S. aureus and Escherichia coli (together accounting for over 40% of BSIs), followed by Klebsiella pneumoniae, P. aeruginosa, and Enterococcus faecalis (see Table 1). Notably, the number of E. coli isolates increased (from 18.7% in 1997 to 2000, to 24.0% in 2013 to 2016) whereas the number of S. aureus isolates declined (from 22.5% to 18.7%), as an overall proportion of all BSI. This change was accompanied by an increase in the proportion of GNB among the top 10 pathogens causing BSI (from 33.5% to 43.4% between the years 1997 to 2000 and the years 2013 to 2016). The proportion of Streptococcus pneumoniae isolates declined from 4.2% of all BSI in 1997 to 2000 to less than 2.0% of all BSI in 2009 to 2016.

**TABLE 1:** Rank order of pathogens causing bloodstream infection worldwide in the SENTRY Program by 4-year period

Rank	Pathogen (%) during:					
	1997–2000	2001–2004	2005–2008	2009–2012	2013–2016	All yrs
1	S. aureus (22.5)	S. aureus (22.7)	E. coli (20.0)	E. coli (21.3)	E. coli (24.0)	S. aureus (20.7)
2	E. coli (18.7)	E. coli (20.2)	S. aureus (19.4)	S. aureus (18.8)	S. aureus (18.7)	E. coli (20.5)
3	K. pneumoniae (6.8)	K. pneumoniae (6.6)	K. pneumoniae (7.8)	K. pneumoniae (8.5)	K. pneumoniae (9.9)	K. pneumoniae (7.7)
4	P. aeruginosa (5.1)	E. faecalis (5.6)	P. aeruginosa (5.4)	E. faecalis (5.3)	P. aeruginosa (5.4)	P. aeruginosa (5.3)
5	E. faecalis (5.0)	P. aeruginosa (5.4)	E. faecalis (5.1)	P. aeruginosa (5.2)	E. faecalis (5.0)	E. faecalis (5.2)
6	S. epidermidis (4.8)	S. epidermidis (3.9)	S. epidermidis (3.4)	E. faecium (3.8)	S. epidermidis (4.1)	S. epidermidis (3.8)
7	S. pneumoniae (4.2)	S. pneumoniae (3.5)	E. cloacae (3.3)	S. epidermidis (3.1)	E. faecium (3.4)	E. cloacae (2.9)
8	E. cloacae (2.9)	E. cloacae (3.1)	E. faecium (3.1)	E. cloacae (2.8)	E. cloacae (2.1)	S. pneumoniae (2.8)
9	E. faecium (1.7)	E. faecium (2.2)	A. baumannii^*a*^ (2.4)	A. baumannii (2.4)	A. baumannii (2.0)	E. faecium (2.8)
10	S. agalactiae (1.5)	P. mirabilis (1.7)	S. pneumoniae (2.2)	S. pneumoniae (1.9)	S. pneumoniae (1.9)	A. baumannii (2.0)

a Acinetobacter baumannii-Acinetobacter calcoaceticus species complex.

Pathogen frequency varied somewhat over time and by region, hospital-onset (HO) or community-onset (CO) status, and age (see Tables 1, 2, 3, and 4). However, S. aureus and E. coli remained predominant, with S. aureus representing a larger proportion of BSIs in North and Latin America (24.5% and 20.1% overall, respectively), while E. coli was predominant in Europe and the Asia-Pacific region (24.1% and 26.0% overall, respectively). Major decreases in the frequency of S. aureus detection occurred in Latin America (from 21.5% in 1997 to 2000 to 16.4% in 2013 to 2016) and the Asia-Pacific region (from 20.8% to 13.9%; Table 2). S. pneumoniae frequency decreased during the study period, especially in Latin America (from 4.0% in 1997 to 2000 to 0.4% in 2013 to 2016) and the Asia-Pacific region (from 4.6% to 0.9%). In contrast, E. coli and K. pneumoniae frequencies increased in all regions, with the greatest increases in Europe and the Asia-Pacific regions (Table 2). Acinetobacter spp. represented higher proportions of BSI in Latin America and the Asia-Pacific regions (4.4% and 3.2% overall, respectively) than elsewhere.

**TABLE 2:** Rank order and frequency of most common organisms causing bloodstream infections in the 1997-to-2000 and 2013-to-2016 time periods stratified by region

Rank^*a*^	Frequency of species in the first (1997–2000) and last (2013–2016) time periods for:			
	North America (% during 1997–2000, % during 2013–2016)	Latin America (% during 1997–2000, % during 2013–2016)	Europe (% during 1997–2000, % during 2013–2016)	Asia-Pacific (% during 1997–2000, % during 2013–2016)
1	S. aureus (25.3, 24.3)	E. coli (17.2, 18.3)	E. coli (21.0, 27.0)	E. coli (21.6, 33.7)
2	E. coli (17.5, 19.8)	S. aureus (21.5, 16.4)	S. aureus (18.2, 16.9)	S. aureus (20.8, 13.9)
3	K. pneumoniae (6.5, 8.6)	K. pneumoniae (9.2, 13.6)	K. pneumoniae (5.8, 10.1)	K. pneumoniae (7.6, 13.5)
4	E. faecalis (6.2, 5.4)	P. aeruginosa (6.5, 7.1)	P. aeruginosa (5.9, 5.8)	P. aeruginosa (4.8, 5.7)
5	P. aeruginosa (4.5, 4.8)	E. cloacae (3.6, 5.9)	E. faecalis (4.6, 5.4)	E. cloacae (3.4, 3.0)
6	S. epidermidis (3.3, 4.6)	A. baumannii^*b*^ (3.2, 5.5)	S. epidermidis (7.8, 4.1)	E. faecalis (3.4, 2.9)
7	E. faecium (2.3, 3.4)	S. epidermidis (4.6, 5.4)	E. faecium (1.5, 4.0)	A. baumannii^*b*^ (2.1, 2.7)
8	E. cloacae (2.8, 3.1)	E. faecalis (2.2, 5.0)	E. cloacae (2.7, 2.6)	E. faecium (1.1, 2.6)
9	S. pneumoniae (4.8, 2.4)	S. marcescens (1.5, 3.3)	A. baumannii^*b*^ (1.8, 2.4)	S. epidermidis (4.8, 2.5)
10	S. agalactiae (2.0, 2.2)	E. faecium (0.3, 2.4)	P. mirabilis (1.8, 2.3)	S. agalactiae (1.2, 1.9)

a Rank order based on the 2013-to-2016 time period.

b Acinetobacter baumannii-Acinetobacter calcoaceticus species complex.

**TABLE 3:** Rank order of pathogens causing bloodstream infection worldwide submitted to the SENTRY Program, 1997–2016, by age group

Rank	Pathogen (%) for patients aged:					
	<1 yr	1–5 yrs	6–18 yrs	19–49 yrs	50–64 yrs	>64 yrs
1	S. aureus (16.4)	S. aureus (15.9)	S. aureus (26.4)	S. aureus (24.9)	S. aureus (23.1)	E. coli (26.6)
2	E. coli (13.7)	S. pneumoniae (11.4)	E. coli (12.6)	E. coli (18.1)	E. coli (19.9)	S. aureus (20.1)
3	K. pneumoniae (8.6)	E. coli (9.2)	P. aeruginosa (6.6)	K. pneumoniae (7.3)	K. pneumoniae (8.6)	K. pneumoniae (8.0)
4	E. faecalis (6.9)	K. pneumoniae (7.9)	K. pneumoniae (6.5)	P. aeruginosa (5.4)	P. aeruginosa (5.9)	E. faecalis (5.9)
5	S. epidermidis (6.3)	P. aeruginosa (5.7)	S. epidermidis (5.1)	E. faecalis (4.8)	E. faecalis (5.3)	P. aeruginosa (5.4)

**TABLE 4:** Rank order of pathogens causing BSI worldwide submitted to the SENTRY Program, 1997–2016, by community or hospital onset

Rank	Pathogen (%)	
	Community onset (*n* = 102,638)	Hospital onset (*n* = 103,945)
1	E. coli (26.6)	S. aureus (21.3)
2	S. aureus (22.4)	E. coli (15.6)
3	K. pneumoniae (7.2)	K. pneumoniae (8.8)
4	S. pneumoniae (5.2)	P. aeruginosa (7.4)
5	E. faecalis (4.7)	E. faecalis (6.4)
6	P. aeruginosa (3.7)	S. epidermidis (4.8)
7	E. cloacae (2.4)	E. faecium (4.3)
8	S. agalactiae (2.3)	E. cloacae (4.0)
9	S. epidermidis (2.2)	A. baumannii^*a*^ (3.2)
10	P. mirabilis (2.0)	S. marcescens (2.1)

a Acinetobacter baumannii-Acinetobacter calcoaceticus species complex.

While E. coli and S. aureus were the two most common causes of BSI in both HO-BSI and CO-BSI, they were much more predominant for CO-BSI (57.8% versus 31.3% for HO-BSI). HO-BSI was more often due to non-E. coli GNB (P. aeruginosa and Acinetobacter spp.; Table 4).

**FIG 1:**
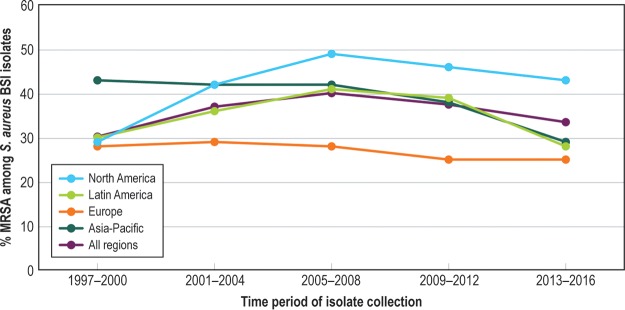
Twenty-year trend in percent methicillin-resistant S. aureus (MRSA) among all S. aureus bloodstream infections, SENTRY, 1997 to 2016.

**FIG 2:**
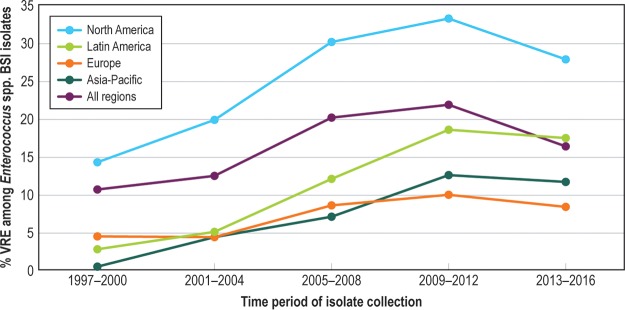
Twenty-year trend in percent vancomycin-resistant enterococci (VRE) among *Enterococcus* sp. bloodstream infections, SENTRY, 1997 to 2016.

The prevalence of ORSA increased until 2005 to 2008 and then declined in all regions and among HO-BSI and CO-BSI (Fig. 1). Of the 56,575 S. aureus BSI isolates tested against vancomycin, only 1 had a vancomycin MIC in the CLSI nonsusceptible category (8 mg/liter), and no trend toward an increase in the vancomycin MIC over time (“MIC creep”) was detected. The proportion of VRE (vancomycin MIC, >4 mg/liter) declined after 2012 and was 16.4% overall (see Fig. 2). Daptomycin resistance among S. aureus and enterococci remained rare (<0.1%).

**FIG 3:**
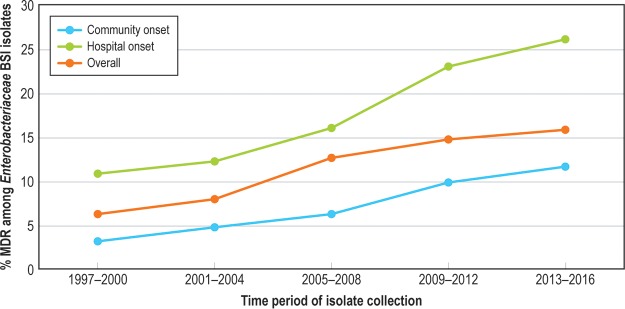
Twenty-year trend in percent multidrug resistance (MDR) among Enterobacteriaceae bloodstream infection, by community-onset versus hospital-onset infection, SENTRY, 1997 to 2016.

**FIG 4:**
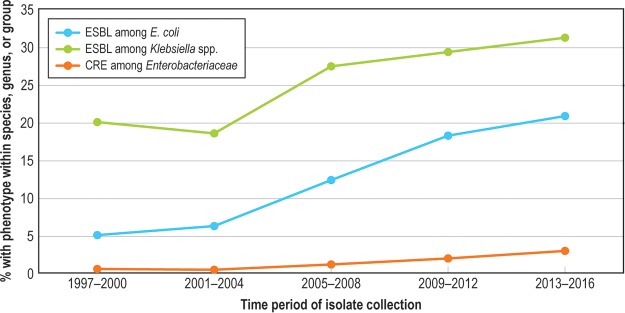
Twenty-year trend in percent extended-spectrum-β-lactamase (ESBL)-resistant and carbapenem-resistant Enterobacteriaceae (CRE) among selected Enterobacteriaceae, SENTRY, 1997 to 2016.

The frequency of MDR Enterobacteriaceae among all Enterobacteriaceae isolates increased from 6.2% in 1997 to 2000 to 15.8% in 2013 to 2016 and was highest among the HO-BSI isolates (Fig. 3). The frequency of MDR among Enterobacteriaceae varied by region, with the highest rates observed in Latin America (28.1%). ESBL-phenotype E. coli, ESBL-phenotype *Klebsiella* spp., and CRE phenotypes also increased over the surveillance period (Fig. 4). MDR rates were highest among the nonfermentative GNB (P. aeruginosa at 26.3% and Acinetobacter baumannii-Acinetobacter calcoaceticus complex at 70.6%); 48 isolates of Acinetobacter baumannii-Acinetobacter calcoaceticus (0.9%) and 9 isolates of P. aeruginosa (<0.1%) were pan-drug resistant. Colistin was the only agent with predictable activity against Acinetobacter baumannii-Acinetobacter calcoaceticus complex (96.9% susceptible; Table 5).

**TABLE 5:** Activity of antimicrobial agents tested against Staphylococcus aureus, Enterobacteriaceae, and Acinetobacter baumannii-Acinetobacter calcoaceticus species complex submitted to the SENTRY Program, 1997–2016

Organism/antimicrobial agent (no. tested)	MIC_50_ (mg/liter)	MIC_90_ (mg/liter)	CLSI^*a*^			EUCAST^*a*^		
			%S	%I	%R	%S	%I	%R
Staphylococcus aureus (56,579)								
Ceftaroline (16,658)	0.25	1	96.2	3.7	0.1	96.2	3.7	0.1
Ceftobiprole (23,214)	0.5	2				99.4		0.6
Dalbavancin (36,161)	0.06	0.06	>99.9^*b*^			99.7		0.3
Daptomycin (37,814)	0.25	0.5	99.9			99.9		0.1
Linezolid (53,595)	2	2	>99.9		<0.1	>99.9		<0.1
Teicoplanin (56,570)	≤2	≤2	>99.9^*c*^			98.8		1.2
Tigecycline (37,085)	≤0.12	0.25	99.8^*b*^			99.8		0.2
Vancomycin (56,575)	1	1	99.9	0.1	0.0	99.9		0.1

Enterobacteriaceae (107,617)								
Amikacin (107,561)	≤4	≤4	97.4	1.3	1.3	95.5	2.0	2.6
Ampicillin-sulbactam (74,048)	16	>16	49.4	17.1	33.5	49.4		50.6
Aztreonam (107,580)	≤0.12	16	86.5	1.6	11.9	84.0	2.5	13.5
Cefepime (107,581)	≤0.5	4	89.3	3.0^*d*^	7.7	87.5	3.3	9.1
Ceftazidime-avibactam (31,672)	0.12	0.25	99.7		0.3	99.7		0.3
Ceftriaxone (107,575)	≤0.25	>8	83.5	0.8	15.7	83.5	0.8	15.7
Ciprofloxacin (107,567)	≤0.5	>2	80.9	1.7	17.5	76.6	1.9	21.5
Colistin (54,476)	≤0.5	>4				88.0		12.0
Doxycycline (59,912)	2	>8	66.5	9.1	24.5			
Gentamicin (107,561)	≤2	>8	87.9	1.1	11.0	86.7	1.2	12.1
Imipenem (107,322)	≤0.5	1	95.2	3.1	1.7	98.3	1.1	0.6
Levofloxacin (107,571)	≤0.5	>4	82.5	2.4	15.1	79.2	2.3	18.5
Meropenem (107,529)	≤0.12	≤0.12	98.8	0.2	1.0	99.0	0.3	0.7
Minocycline (56,100)	2	>8	77.2	8.5	14.3			
Piperacillin-tazobactam (107,301)	2	16	90.0	4.4	5.6	86.8	3.2	10.0
Tetracycline (107,577)	≤4	>8	64.0	2.8	33.2			
Tigecycline (68,141)	0.25	1	98.4^*b*^	1.4	0.2	94.9	3.5	1.6
Tobramycin (107,579)	0.5	>8	86.4	3.1	10.5	83.6	2.8	13.6
Trimethoprim-sulfamethoxazole (107,571)	≤0.5	>1	74.7		25.3	74.7^*c*^		

P. aeruginosa (14,562)								
Amikacin (14,559)	≤4	16	90.0	2.4	7.6	86.1	4.0	10.0
Aztreonam (14,558)	8	>16	66.7	13.9	19.4	1.7	78.9	19.4
Cefepime (14,559)	4	>16	79.9	10.0	10.1	79.9		20.1
Ceftazidime (14,557)	≤2	>16	77.7	4.9	17.4	77.7		22.3
Ceftazidime-avibactam (3,911)	2	8	93.4		6.6	93.4		6.6
Ciprofloxacin (14,559)	≤0.5	>2	74.8	3.0	22.2	71.0		29.0
Colistin (7,107)	1	2	99.3		0.7	99.3		0.7
Doripenem (9,112)	0.5	>4	78.9	8.0	13.1	71.5	7.3	21.1
Gentamicin ((14,557)	≤2	>8	79.8	2.8	17.4	79.8		20.2
Imipenem (14,534)	1	>8	75.3	4.7	20.0	80.0	7.2	12.7
Levofloxacin (14,557)	≤0.5	>4	72.9	4.1	23.0	66.2		33.8
Meropenem (14,556)	0.5	>8	77.5	6.2	16.3	77.5	11.8	10.8
Piperacillin-tazobactam (14,549)	8	>64	73.8	11.3	14.9	73.8		26.2
Polymyxin B (8,855)	≤1	2	99.6	0.4	<0.1			
Tobramycin (14,558)	0.5	>8	82.8	0.7	16.6	82.8		17.2

Acinetobacter baumannii-Acinetobacter calcoaceticus complex (5,333)								
Amikacin (5,332)	>32	>32	45.7	4.2	50.1	42.6	3.1	54.3
Ampicillin-sulbactam (4,056)	>16	>16	36.5	11.8	51.7			
Cefepime (5,332)	>16	>16	35.5	11.1	53.4			
Ceftazidime (5,332)	>16	>16	32.8	5.8	61.5			
Ciprofloxacin (5,332)	>2	>2	32.4	0.6	67.0	32.4		67.6
Colistin (3,124)	≤0.5	2	96.9		3.1	96.9		3.1
Doxycycline (3,238)	≤1	>8	67.4	1.5	31.1			
Gentamicin (5,324)	>8	>8	39.0	5.4	55.6	39.0		61.0
Imipenem (5,333)	2	>8	55.3	3.1	41.6	55.3	5.9	38.8
Levofloxacin (5,332)	>4	>4	34.6	8.3	57.1	32.3	1.1	66.7
Meropenem (5,326)	2	>8	52.0	4.1	43.8	52.0	8.9	39.1
Minocycline (3,098)	≤1	>8	81.5	8.4	10.1			
Piperacillin-tazobactam (5,320)	>64	>64	30.8	9.4	59.8			
Tetracycline (5,178)	8	>8	42.4	13.2	44.4			
Tigecycline (3,688)	0.5	2						
Tobramycin (5,330)	4	>8	52.4	3.2	44.4	52.4		47.6
Trimethoprim-sulfamethoxazole (5,331)	>1	>1	41.7		58.3	41.7^*c*^		

a Criteria as published by CLSI (40) and EUCAST (41), the latter for comparison only. S, sensitive; I, intermediate; R, resistant.

b Breakpoints from FDA package insert (42).

c Dilution range did not extend far enough to determine whether the data represented intermediate or resistant status, so only the susceptible percentage is displayed.

d Intermediate data interpreted as susceptible-dose dependent.

The antimicrobial activity of selected agents against the 107,617 Enterobacteriaceae isolates is outlined in Table 5. Over 95% of these BSI pathogens were susceptible *in vitro* to amikacin, ceftazidime-avibactam, carbapenems, and tigecycline.

## DISCUSSION

Many surveillance programs are designed to monitor trends in BSI. However, the scope of most programs is limited to specific patient populations, countries, or regions (5, 7–13) and the programs do not confirm the organism identification or susceptibility data at a reference laboratory. The SENTRY Program performs global surveillance, monitoring pathogens from consecutive BSI episodes and testing all isolates at the central reference laboratory (6). Collecting isolates from consecutive episodes allows inference of rates of prevalence at each site, providing the opportunity to examine large-scale trends.

Major findings from the first 2 decades of SENTRY Program BSI surveillance include (i) the predominance of S. aureus and E. coli as BSI pathogens worldwide, (ii) the decline in the proportion of BSI due to important resistant Gram-positive pathogens (ORSA, VRE, daptomycin resistance among S. aureus and enterococci [DRE]) during the second decade of surveillance, and (iii) the ongoing increase in the detection of GNB and in MDR-GNB as a proportion of all causes of BSI worldwide.

Population-based BSI surveillance is consistent with our findings regarding the prominence of S. aureus and E. coli as BSI pathogens. Laupland (10) has reviewed population-based BSI surveillance programs from various regions, noting that E. coli and S. aureus were the two most common BSI pathogens, with estimated incidence rates of 35 and 25 per 100,000, respectively. By comparison, the next most common BSI cause was S. pneumoniae, at 10 cases/100,000 (10). SENTRY surveillance suggests that E. coli has become slightly more prominent than S. aureus over the past 2 decades, while the proportion of BSI caused by S. pneumoniae has declined. The reasons for these trends are not yet understood but may be related to changes in health care delivery, emergence and global spread of the E. coli sequence type 131 clone (14), increased focus on S. aureus disease prevention in community (15) and health care (16, 17) settings, and the introduction of pneumococcal conjugate vaccines (18). Whatever the reasons, our findings suggest that priority should be given to developing novel prevention approaches for these two important BSI pathogens, including continued research and development of vaccine candidates (19, 20).

For the first decade of SENTRY BSI surveillance, the proportions of important resistant phenotypes among Gram-positive pathogens (ORSA and VRE) were stable or increasing. Somewhat surprisingly, the proportions of S. aureus BSI and *Enterococcus* sp. BSI due to ORSA and VRE, respectively, declined over the past 5 to 10 years of the surveillance period. The decline in the proportion of ORSA that we report is consistent with other regional and national surveillance programs that observed reductions in ORSA infections, or in the proportion of S. aureus that are ORSA, during the decade of the 2000s (7–9, 17, 21). Although this decline has coincided with an increased emphasis on hospital infection prevention practices worldwide, the reasons for it are unclear and likely to be complex and multifactorial (22–26). We discuss this in more detail in a recent report on overall S. aureus infections at all anatomic infection sites in the SENTRY Program (27). We also observed that the percentage of vancomycin resistance among enterococci stabilized and has begun to decline. Given the fact that VRE BSI is an almost exclusively health care-associated infection, it seems likely that a decline in VRE as a proportion of all enterococcal infections is related to improved hospital infection prevention and coincides with general reductions in the rates of health care-associated infections (28). Finally, only one S. aureus BSI isolate with vancomycin resistance (MIC = 8 mg/liter) was detected across 2 decades of surveillance, and daptomycin resistance among indicated S. aureus and *Enterococcus* spp. (MIC, >1 mg/liter) has remained extremely uncommon.

In contrast to the resistance trends seen among Gram-positive pathogens, SENTRY surveillance revealed that detection of GNB is increasing as a proportion of all BSI causes and that the proportion of important GNB resistance phenotypes has increased over the past 2 decades. Our findings support reports of the emergence and spread of ESBL (5, 14, 29, 30) and carbapenemase (31–33) enzymes worldwide. While some of these enzymes, e.g., CTX-M and KPC (14, 31, 32), have disseminated globally, rates of regional (and even local) variations in prevalence are substantial (33). This increases the importance of developing regional surveillance and prevention collaborations in an attempt to interrupt transmission in communities and across health care networks (34, 35). Beyond Enterobacteriaceae, the most resistant GNB in our surveillance were the nonfermenters P. aeruginosa and Acinetobacter spp. These are the species among which the pan-drug-resistant GNB are most likely to be detected (36) and for which new drug development is increasingly critical (37, 38).

The surveillance data we present in this report have limitations. As a sentinel network that collects pathogens from selected medical centers, the SENTRY Program does not provide population-based information about the incidence of infections in a given region. In addition, not all sentinel medical centers participated in each year of the 20-year surveillance program. As participating centers leave the program, additional centers from that region are added, with the goal of maintaining robust and broadly representative samples from as many countries and regions as possible. Furthermore, regions of the world with limited resources for clinical laboratory support, e.g., Africa, are also underrepresented or not represented in this report. Finally, while we have investigated some of the trends noted, we do not present our molecular or sequencing data in this report.

Nonetheless, the duration and global scope of the SENTRY Program provide important insights into trends in pathogen frequency and antimicrobial resistance trends among BSI pathogens. While E. coli and S. aureus remain the major causes of BSI worldwide, there is an important divergence between the antimicrobial resistance challenges posed by Gram-positive and Gram-negative bacterial causes of BSI. While the most important resistance phenotypes among Gram-positive BSI pathogens are stable or declining as a proportion of reported infections, the proportion of MDR GNB is increasing worldwide. Improved diagnostic, therapeutic, and preventive approaches are urgently needed for these pathogens. Ongoing global surveillance remains important to help inform development, implementation, and follow-up of these approaches.

## MATERIALS AND METHODS

### Organism collection.

SENTRY is a sentinel surveillance program for tracking antimicrobial resistance worldwide via a global network of medical centers. Each participating SENTRY Program center submitted bacterial isolates and clinical data for consecutive unique episodes of BSI each month during the surveillance period (one isolate per patient, from any acute care setting). Isolate identification was confirmed at the central reference laboratory (JMI Laboratories, North Liberty, IA, USA) using conventional and proteomic methods. This report describes the organism distribution and antimicrobial susceptibility trends among the 264,901 BSI isolates collected from 238 SENTRY participating centers in North America (72 centers in the United States and Canada), Latin America (18 centers in 7 countries), Europe (65 centers in 23 countries), and the Asia-Pacific region (83 centers in 12 countries) between January 1997 and December 2016. When the sample collection date was 3 or more days after the admission date, we designated the BSI episode a “hospital-onset” episode.

### Resistance phenotypes.

VRE was defined on the basis of a vancomycin MIC of >4 mg/liter (nonsusceptible per CLSI and resistant per the European Committee on Antimicrobial Susceptibility Testing [EUCAST]). CRE was defined as resistance to meropenem, imipenem (not applied for Proteus mirabilis or indole-positive *Proteeae*), and/or doripenem. The ESBL phenotype was defined as E. coli, P. mirabilis, and *Klebsiella* spp. with an aztreonam, ceftazidime, or ceftriaxone MIC of ≥2 mg/liter. MDR among GNB was defined using CDC criteria (nonsusceptibility to at least one drug in ≥3 of the following antibiotic classes: broad-spectrum cephalosporins, carbapenems, broad-spectrum penicillin combined with a β-lactamase inhibitor, fluoroquinolones, aminoglycosides, glycylcyclines (for Enterobacteriaceae only), and the polymyxins (36). Pan-drug resistance was defined as nonsusceptibility to a drug in all antibiotic classes.

### Susceptibility methods.

Susceptibility testing was performed against more than 20 antimicrobial agents, using reference broth microdilution methods and interpretive MIC breakpoints as described by CLSI (39, 40) and EUCAST (41). FDA breakpoints were used if CLSI breakpoints were not available. Quality control was performed as recommended by CLSI, and results were all within established ranges.
